# Unusual case presentation of intestinal *Sarcocystis hominis* infection in a healthy adult

**DOI:** 10.1099/jmmcr.0.T00019

**Published:** 2014-12-01

**Authors:** Laila Nimri

**Affiliations:** Department of Medical Laboratory, Jordan University of Science and Technology, Irbid , Jordan

**Keywords:** beef shawarma, intestinal, non-bloody diarrhea, *Sarcocystis hominis*, Sarcocystosis, severe case

## Abstract

**Introduction:**

Sarcocystosis is mainly a veterinary problem; however, humans can serve as the definitive host for at least two species (*Sarcocystis hominis* and *Sarcocystis suihominis*). Intestinal infections occur in the definitive host after ingesting the intramuscular cysts (sarcocysts) in the intermediate host, which initiate sexual stages in the intestine that terminate in oocysts excreted in the faeces.

**Case presentation:**

A 19-year-old male presented with diffuse abdominal pain, watery non-bloody diarrhoea, nausea, vomiting and intermittent low-grade fever that lasted for more than 3 weeks. Multiple stool cultures on enriched and selective media gave negative results. Microscopic examination of wet mounts of stool prepared from formalin/ethyl acetate concentrates, together with permanent staining helped in making a definitive diagnosis and ruling out other coccidian parasites. Diagnosis of the parasite as *S. hominis* was made based on the size and morphology of the individual sporocysts that were observed in the wet-mount preparations. This severe case of intestinal sarcocystosis in a healthy adult after eating undercooked beef shawarma meat is described.

**Conclusion:**

The unusual presentation of intestinal sarcocystis described in this case is very rare. The clinical signs and size and morphology of both oocysts and sarcocysts observed in concentrated wet mounts of stool helped in the definitive diagnosis. The food ingested prior to the appearance of symptoms was important in making the definitive diagnosis of the parasite as *S. hominis*, as well as the incubation period and treatment.

## Introduction

*Sarcocystis* spp. are intracellular protozoan parasites in the phylum Apicomplexa. These parasites have an indirect life cycle that includes both definitive and intermediate hosts. Intestinal infections occur in the definitive host after ingesting the cysts (sarcocysts) in muscles of the intermediate host, which initiate sexual stages in the intestine that ends in excreting oocysts in the faeces. Tissue invasion due to the development of asexual stages and the formation of intramuscular cysts occurs in the intermediate hosts after ingesting either oocysts or sporocysts from the environment. Sarcocystosis due to various *Sarcocystis* spp. is mainly a veterinary problem, as more than 50 % of cattle, pigs and sheep are infected (Acha & Szyfres, 2003). However, two species are known to be pathogenic to humans: *Sarcocystis hominis* uses cattle as the intermediate host, whilst *Sarcocystis suihominis* uses pig as the intermediate host. Humans can serve as the definitive host after ingesting raw or undercooked meat of the intermediate hosts (Fayer, 2004). Humans can also act as an intermediate host after ingesting food or water contaminated with oocysts excreted in the faeces of an infected animal.

Although *Sarcocystis* spp. are considered a clinically important human intestinal protozoan (Farthing, 2006), the prevalence of intestinal sarcocystosis is low and often subclinical, except in volunteers who ingest large numbers of sarcocysts (Fayer, 2004). Data on the prevalence of human intestinal sarcocystosis is limited, and most infections reflect case reports. Consequently, many infections may go unreported, but infections are certainly more frequent in Europe and Thailand than in other countries (Dubey *et al*., 1989). *Sarcocystis* spp. were observed in faecal specimens examined from children in Poland (10.4 %) and in Germany (7.3 %). In addition, 1.1 % of 1228 Vietnamese who worked in Central Slovakia in 1987–1989 were positive for *Sarcocystis* (Straka *et al*., 1991), and 23.2 % of stool samples examined from Thai workers were found to be positive for *Sarcocystis* spp. (Wilairatana *et al*., 1996).

The prevalence of intestinal sarcocystosis in humans is probably underestimated, as oocysts are often present in small numbers and are rarely detected in stool. The oocyst wall is thin, with two adjacent sporocysts, each containing four sporozoites. Intact oocysts usually appear in stool only in the first few days of patency. Generally, the delicate oocyst wall ruptures while still in the intestine, often releasing individual sporocysts that pass into the faeces (Zeibig, 2013), which are often the only stage observed in the faeces (Fayer, 2004). Intestinal infections in humans are generally unreported because they are rarely associated with illness.

Here, a severe case of intestinal *S. hominis* infection observed in an otherwise healthy adult is described.

## Case report

A 19-year-old male presented with diffuse abdominal pain, watery non-bloody diarrhoea, nausea, vomiting and intermittent low-grade fever. The patient had experienced these symptoms during the preceding weeks.

**Fig. 1.**
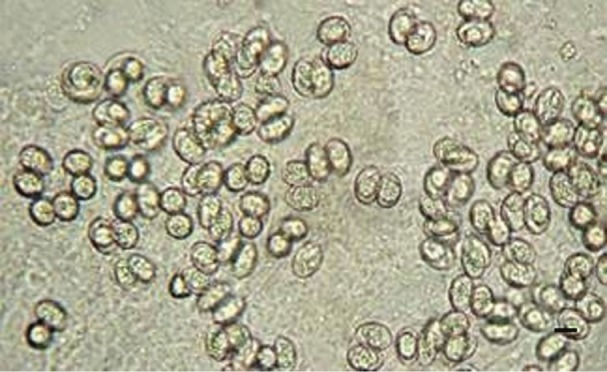
*S. hominis* individual sporocysts and oocysts, each containing two sporocysts. Wet mount; bar, 12 µm.

Prior to treatment with conventional antibiotics, multiple stool cultures on blood agar, MacConkey agar (Oxoid) and other selective media in addition to other tests for enteropathogens (bacterial and viral) gave negative results. Microscopic examinations of fresh stool specimens and of wet-mount concentrates from flotation and sedimentation techniques were performed. A multiple stool ova and parasites examination of wet mounts and permanently stained smears with trichrome, iron haematoxlyin and modified Kinyoun acid-fast stain were prepared from formalin/ethyl acetate concentrates. This case was initially suspected to be *Cryptosporidium* gastroenteritis because of the severity of symptoms or *Cyclospora cayetanensis* based on the oocyst size of 8–10 µm, which is close to the size of *Sarcocystis* sporocysts. The untypical presentation and duration of the symptoms, especially the diarrhoea lasting more than 3 weeks, complicated and delayed the diagnosis of the case. The parasite’s individual sporocysts were initially misidentified by the laboratory. This error would not have affected the patient treatment for this parasite if this was a mild self-limiting case. However, the definitive diagnosis of the parasite as *S. hominis* was made based on the microscopic demonstration of the individual sporocysts (mean size 9×14 μm) and oocysts (mean size 17×15 μm), each containing two sporocysts in the wet-mount preparations (Fig. 1).

Based on the history of the patient, symptoms started approximately 4 h after eating undercooked beef meat in shawarma sandwiches, which is a very unusual short incubation period. The symptoms, which lasted for 6 weeks from their first appearance, were resolved after a 10-day course of trimethoprim-sulfamethoxazole (co-trimoxazole), and stool specimens tested negative for the sporocysts after treatment.

## Discussion

Presumptive diagnosis of human intestinal sarcocystosis is based on symptoms and a history of recently having eaten raw or undercooked meat. However, the definitive diagnosis requires the identification of oocysts/sporocysts in a patient’s faeces. Intestinal sarcocystosis in immunocompetent individuals is often asymptomatic, very mild or subclinical, and is self-limiting (Fayer, 2004).

This case represents an unusual presentation of intestinal *S. hominis* infection in an otherwise healthy adult. From the history of the patient, the source of infection was traced back to eating undercooked beef meat in several shawarma sandwiches (donairs). The short incubation period of approximately 4 h and the severity of symptoms might be explained by ingesting large numbers of *Sarcocystis* tissue sarcocysts in the undercooked beef.

The early clinical signs of intestinal sarcocystis have been reported previously to occur as early as 3–6 h after ingestion in human volunteers (Acha & Szyfres, 2003), which is similar to this case.

A study found that 100 % of raw kibbe samples from local restaurants in Sao Paulo were positive for *Sarcocystis* infection (Pena *et al.*, 2001). Kibbe is the national dish in Lebanon and consists of freshly minced raw meat (lamb or beef) served with spices. The symptoms of intestinal infection reported by volunteers who consumed raw kibbe support the fact that intestinal *Sarcocystis* infections occur in areas where ingestion of raw or undercooked meat is common.

Infections with *Sarcocystis* spp. can be prevented by thorough cooking or freezing meat to kill bradyzoites in the tissue cysts.

In conclusion, the diagnosis of gastrointestinal pathogens from stools of patients with severe diarrhoea requires not only culture for bacterial pathogens but the routine inclusion of concentration techniques and microscopy to identify possible parasites. This case will increase awareness in clinical laboratories of the fact that *Sarcocystis* spp. can induce severe diarrhoea, even in otherwise healthy individuals.
